# Long-Term Disability Outcomes in Relapsing–Remitting Multiple Sclerosis Patients: Impact of Clinical and Demographic Factors on Disease Progression

**DOI:** 10.3390/jcm13061813

**Published:** 2024-03-21

**Authors:** Laura Barcutean, Smaranda Maier, Zoltan Bajko, Adina Stoian, Oana Mosora, Emanuela Sarmasan, Ion-Bogdan Manescu, Rodica Balasa

**Affiliations:** 1 Department of Neurology, George Emil Palade University of Medicine, Pharmacy, Science, and Technology of Targu Mures, 540142 Targu Mures, Romania; laurabarcutean@gmail.com (L.B.); zoltan.bajko@umfst.ro (Z.B.); rodica.balasa@umfst.ro (R.B.); 2 Neurology 1 Clinic, Emergency Clinical County Hospital, 540136 Targu Mures, Romania; cretadina@yahoo.com (A.S.); oanamosora.92@yahoo.com (O.M.); sarmasan.emanuela@gmail.com (E.S.); 3 Department of Pathophysiology, George Emil Palade University of Medicine, Pharmacy, Science, and Technology of Targu Mures, 540142 Targu Mures, Romania; 4 Department of Laboratory Medicine, George Emil Palade University of Medicine, Pharmacy, Science, and Technology of Targu Mures, 540139 Targu Mures, Romania; bogdan.manescu@umfst.ro

**Keywords:** multiple sclerosis, progression, relapsing–remitting, secondary-progressive, EDSS

## Abstract

**Background:** Multiple sclerosis (MS) is a prevalent chronic inflammatory and neurodegenerative disease of the central nervous system. The main evolving forms, relapsing–remitting MS (RRMS) and secondary progressive MS (SPMS), lack clear delineation. **Methods:** We conducted an observational study on 523 Caucasian RRMS patients receiving first-line disease-modifying therapies (DMTs), analyzing demographic, clinical, and geographical data. **Results:** RRMS patients experienced a statistically significant reduction in relapse rates post-DMT initiation. Significant differences in time to reach an Expanded Disability Status Score (EDSS) of 3.0 and 6.0 were observed based on demographics and onset topography. Kaplan–Meier analysis revealed that the onset with optic or supratentorial symptoms is linked to a longer time until EDSS = 3.0 is reached. Urban origin correlated with a prolonged time until EDSS = 3.0. Gender and environment showed no significant associations with the hazard of reaching an EDSS = 6.0. Cox regression analysis revealed no significant impact of relapses on the time to reach EDSS scores of 3.0 and 6.0 in our study cohort. **Conclusions:** Multivariate analysis identified several predictive factors for disability progression, including environment, age at onset, and disability level at DMT initiation.

## 1. Introduction

Multiple sclerosis (MS) is the most prevalent chronic inflammatory and neurodegenerative disease of the central nervous system (CNS), affecting over 2 million people worldwide [1,2,3]. In 2020, the Atlas of MS reported a global diagnosis of approximately 2.8 million individuals with MS, marking a 30% increase from the 2013 data [4]. Over time, a significant portion of individuals with MS transition to a progressive clinical course, leading to impaired mobility and cognition [5].

The classification of the evolving forms of MS has undergone systematic refinement over the past decades, aimed at stratifying distinct MS subtypes and establishing clear guidelines for treatment and monitoring. The definitions of classical phenotypes such as relapsing–remitting MS (RRMS), secondary progressive MS (SPMS), and primary progressive MS (PPMS) [6,7,8], widely used in clinical practice, were amended in 2013 by Lublin et al. The Committee incorporated disease activity and progression into the diagnostic process. This included new relapses or progression (according to the Expanded Disability Status Scale (EDSS)), emerging or enlarging T2 lesions, or active lesion in brain and spinal cord imaging [7,9,10]. In 2017, the updated McDonald criteria were proposed for both clinical and research practice [11]. In 85% of cases, the clinical course at onset is RR [7,11], and patients transition to SPMS within one to two decades following onset [12,13,14,15].

The transition from RR to SPMS presents a major challenge for treatment decision [16]. Disability can result from acute relapses in RRMS, known as relapse-associated worsening, but can also manifest independently of relapses, known as progression independent of relapse activity [17,18,19]. The relationship between relapse occurrence and disability accumulation or progression remains under debate, with some studies indicating no association between relapses and disease progression [18,20,21]. Prior studies delineated two distinct phases of evolution, spanning from onset to reaching an EDSS of 3.0 and 6.0, respectively. The findings demonstrated significant time variability in the evolution of phase 1 (from onset to EDSS 3.0), while phase 2 (from EDSS 3.0 to 6.0) remained consistent. This proposed a two-stage nature of MS, with the initial stage influenced by focal inflammation and the subsequent stage marked by disability progression independent of inflammation [22].

But, recent advances in understanding disability progression in MS have explored the need for a mechanism-driven framework focusing on a biologically based definition of MS progression [23]. Both inflammatory and neurodegenerative processes are present at the onset of the disease. Early cortical damage explained by meningeal inflammation and subpial grey matter demyelination appear to be predictor factors for the degree of progression in comparative MS studies [24]. The pattern of neurodegeneration is either secondary to an acute injury (axonal fragmentation secondary to ongoing inflammation) or retrograde neurodegeneration (which stems from deeper cortical layers and normal-appearing cortex adjacent to subcortical white matter plaques) [25]. The trajectory of disability progression in MS defies a dichotomous classification from the onset, with studies suggesting that even in RRMS populations, most of the quantifiable physical disability can occur independently of relapses [5,19,26,27]. This can stem from patient-specific factors such as sex, age, social and environmental determinants, genetic predisposition, and disease duration. Among the extensively studied clinical predictors of disease progression are the type of onset based on the affected functional system, baseline disability assessed by EDSS, age at onset, living environment, and relapse frequency [28,29,30,31].

## 2. Materials and Methods

### 2.1. Objectives

The aim of this study is to assess the clinical prognostic factors linked to progression in a Caucasian MS population initially displaying a RRMS phenotype and undergoing treatment with first line disease-modifying therapies (DMTs).

### 2.2. General Methodology

We conducted an observational, prospective study that included all the PwMS registered at the Regional Multiple Sclerosis Center in Targu Mures, Neurology 1 Clinic, treated with first-line DMTs. The inclusion criteria were: (1) patients diagnosed with RRMS at onset, according to available guidelines (all patients underwent screening, as per McDonald 2017 criteria [11]); (2) had an RRMS phenotype at DMT initiation (between April 2000 and January 2022); (3) were actively treated (from the onset) with a first-line DMT (interferon, glatiramer acetate, teriflunomide) [32,33,34]; (4) consented to the processing of clinical and demographic data for research purposes. The exclusion criteria were: (1) patients with a different clinical form of MS at onset (PP, SP); (2) patients treated with any other DMTs; (3) patients entering follow-up and treatment with a progressive form of the disease; (4) no immunomodulatory treatment; (5) less than one year of treatment. Dimethyl fumarate, considered a first-line DMT [35], was initiated in our center starting in January 2022; therefore, it was not included in the present analysis. The study included all patients that fit the inclusion and exclusion criteria, and data were analyzed during April 2022–July 2023.

The Neurology 1 Clinic at the Emergency Clinical County Hospital of Targu Mures became the first MS center in Romania to provide DMT to PwMS, beginning in April 2000. Since then, 1377 PwMS have been diagnosed, treated, and monitored as part of the National MS program. Up until the time of this study’s inclusion, a total of 230 patients had withdrawn (due to transfers to neighboring MS centers or abroad, cessation of DMT, or death). In April 2022, our clinic was actively monitoring 634 PwMS, among whom 94 were undergoing treatment with alternative DMTs and 17 had PPMS.

### 2.3. Patient Evaluation

The present study included all PwMS actively treated with standard DMTs and had an RR form at the moment of DMT initiation. We refer to standard DMTs as follows: (a) interferons (IFN) (IFN beta 1b with subcutaneous administration, IFN beta1a with subcutaneous and intramuscular administration); (b) glatiramer acetate (subcutaneous administration); (c) teriflunomide (oral). In order to simplify the presented data, the DMTs were further classified as injectable and oral.

All included patients were assessed based on clinical and demographic data: gender, environment (urban, rural), age, medical history: first neurological symptom, moment of diagnosis, current disease phenotype (RRMS, SPMS), treatment duration, number of relapses before and after treatment initiation. Transition to SPMS was made according to the attending neurologist’s decision according to the 2017 McDonald criteria [11], as all patients were followed longitudinally since the moment of DMT initiation per internal protocols (clinical assessment every six months). The data were collected from the internal database comprising entries detailing each patient’s diagnosis, treatment regime, clinical assessment, and disease evolution. The database is electronically managed, with individual sheets updated regularly by the attending neurologists to reflect the latest information pertaining to each patient’s visit. The cut-off for data entry was January 2022.

A relapse is defined as a distinct and objective significant aggravation of clinical symptoms or the emergence of new neurological symptoms lasting beyond 24 h. For an event to be classified as a relapse, it must occur after a minimum period of 30 days from a previous exacerbation, in the absence of fever or infection [7,11]. The patient-specific values of relapses were calculated using the person-years method and defined as the annual relapse ratio (ARR), with ARR_0: before treatment, ARR_1: during treatment, and ARR_T: entire disease duration. For the COX regression analysis, we used the absolute number of relapses before treatment, denoted as R_0. Onset was not considered as a clinical relapse.

Then, a neurological assessment was conducted based on the calculation of the EDSS scale by the attending neurologists. The EDSS score represents a standardized, objective method for assessing disability in MS [9]. Initial EDSS scores (at the moment of DMT initiation) were extracted from the electronic records of patients. We tracked progression patterns within the analyzed population through rigorous examination of EDSS scores and identified the point at which an EDSS of 3.0 or higher was reached, in the absence of relapses. An EDSS of 3.0 and 6.0 were considered as milestones in disability progression. We defined MEDSS = 3.0 as the moment of reaching an EDSS equal to 3.0 and MEDSS = 6.0 as the moment of reaching an EDSS equal to 6.0.

We selected an EDSS score of 3.0 as the initial disability milestone because it signifies the beginning of mild to moderate irreversible disability. Additionally, an EDSS score of 6.0 was chosen as the subsequent milestone because it marks the first sign of dependence on ambulatory assistance [36,37].

Furthermore, we stratified the time from onset to reaching an EDSS of 3.0, as follows: (A) 0 to 3 years, (B) 3 to 6 years, (C) 6 to 10 years, (D) 10 to 15 years, (E) >15 years.

Onset was evaluated based on the affected CNS region (topography): (1) Supratentorial; (2) Spinal; (3) Optic; (4) Infratentorial; (5) Mixt. Patients’ electronic records were consulted to record the type of onset and the EDSS at the initiation of immunomodulatory treatment (EDSS_0) in our center. For disease progression assessment and COX regression analysis for MEDSS = 3.0 and 6.0, we considered the following disease parameters: (1) age at onset, (2) environment, (3) EDSS_0, (4) R_0, and (5) topography at onset.

### 2.4. Statistical Analysis

The study participants’ clinical and demographic characteristics were assessed through descriptive statistics. Qualitative variable distribution was summarized using absolute and relative frequencies (percentages), while continuous variables were presented using the mean and standard deviation (SD) or median with the associated interquartile range (IQR). The choice between mean/SD and median/IQR depended on the empirical distribution’s fit to the normal probability distribution. The normality of variables was evaluated using the Kolmogorov–Smirnov test, quantile plots (Q-Q plots), as well as skewness and kurtosis (prior to data analysis). If quantitative variable distribution followed a normal probability distribution, parametric tests like the Student’s *t*-test were applied; otherwise, non-parametric tests (Mann–Whitney or Kruskal–Wallis tests with Dunn–Bonferroni correction) were employed.

Kaplan–Meier survival curves were used to estimate the probability of survival until reaching an EDSS score of 3.0 or 6.0, respectively. Multiple Cox regression analyses were employed to determine and quantify the associations between demographic factors and the hazard of reaching an EDSS score of 3.0 or 6.0. These associations were quantified by calculating the relative hazard (HR) and the corresponding 95% confidence interval (95% CI). A significance level of α = 0.05 was applied to all two-tailed statistical tests, and *p*-values < 0.05 were considered statistically significant. Statistical analysis utilized R 4.2.2. statistical computing software and SPSS v.26 for survival analysis.

### 2.5. Ethics Committee

The study was approved by the ethics committee of the Emergency Clinical County Hospital of Targu Mures, no. 13555/21.06.2022.

## 3. Results

### 3.1. Demographic and Clinical Characteristics of the Study Population

The clinical and demographic characteristics of the study cohort are presented in Table 1. A female-to-male ratio of 2.13 was observed, with 359 patients (68.64%) originating from urban areas. The median age at disease onset was 31 years, with a median disease duration of 13 years and a median treatment duration of 9 years. No statistically significant difference was found (*p* = 0.37, Mann–Whitney *U* test) regarding ARR_0 and ARR_1 for the entire patient cohort. However, a statistically significant difference was observed (*p* < 0.001) among PwMS with an RR form at the moment of study inclusion. A total of 200 patients (38.24%) presented with no more relapses after DMT initiation.

Based on the DMT type, 406 (77.62%) of the PwMS were treated with injectable DMTs and 117 (22.37%) with oral DMTs. Immunomodulatory treatment was initiated in 123 cases (23.51%) in the same year as onset, 218 cases (41.68%) within 1–3 years from onset, 114 cases (21.79%) within 4–9 years from onset, and 68 cases (13%) more than 10 years from onset.

In the majority of cases, immunomodulatory treatment was initiated in the same year as the diagnosis for 313 (59.84%) patients, 164 (31.35%) within 1–3 years, and 46 (8.79%) more than 4 years from the diagnosis.

The immunomodulatory treatment line was changed in 171 PwMS (32.69%) during the follow-up period, as follows: clinical-imaging activity 46 (26.90%), progression 52 (30.4%), adverse reaction 48 (28.07%), others 25 (14.61%).Based on the form of MS at study inclusion, 436 PwMS (83.7%) presented with RR form and 87 (16.63%) with SP form. The clinical and demographic data are presented in Table 2.

**Figure 1:**
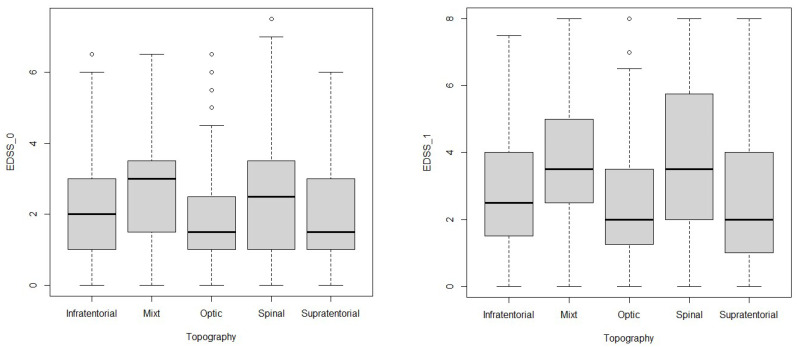
Graphical representations of the onset topography according to EDSS_0 and EDSS_1 (Kruskal–Wallis test with Dunn–Bonferroni corrections, with statistically significant differences emphasized; outlier values shown).

We analyzed the characteristics of the clinical parameters according to the onset topography. Regarding ARR_0 and ARR_T, after using the Dunn–Bonferroni correction, no statistically significant differences were found (adjusted *p* > 0.05). For EDSS_0, a statistically significant difference in the EDSS_0 scores between PwMS with onset through Mixt and Optic topography (adjusted *p* = 0.003), Optic and Spinal (adjusted *p* < 0.001), Mixt and Supratentorial (adjusted *p* = 0.01), and Spinal and Supratentorial (adjusted *p* = 0.004) were found. For EDSS_1, statistically significant differences in EDSS_1 between PwMS with onset through Optic and Spinal topography (adjusted *p* = 0.04) and Spinal and Supratentorial (adjusted *p* = 0.02) were found. Data are summarized in Table 3 and Figure 1.

### 3.2. Kaplan–Meier Survival Analysis for MEDSS = 3.0 and 6.0

Out of the total number of PwMS included in the study (*n* = 523), 259 (49.52%) reached an EDSS of 3.0 or more.

#### 3.2.1. Kaplan–Meier Survival Analysis for MEDSS = 3.0

**Figure 2:**
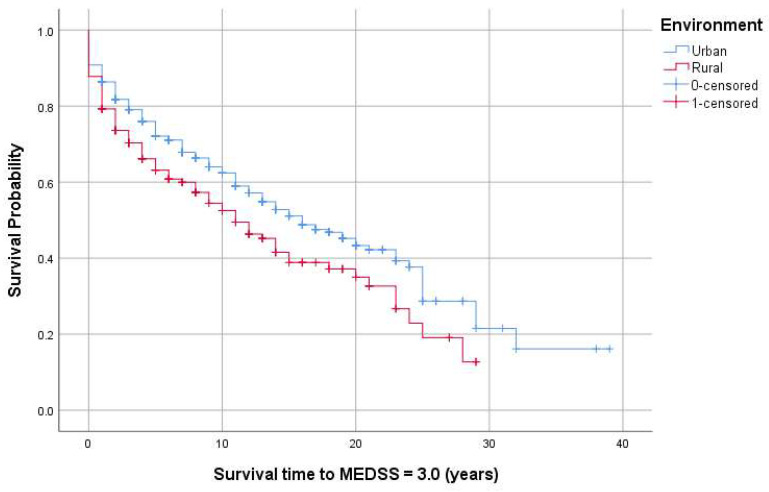
Kaplan–Meier survival curves for MEDSS of 3.0 (measured from onset) based on the environment.

**Figure 3:**
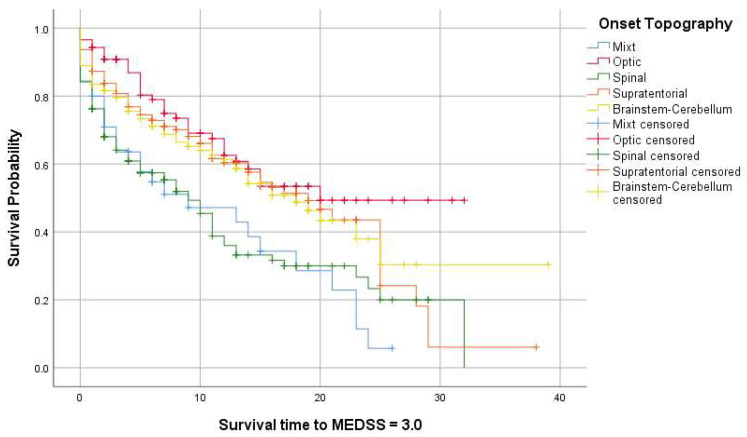
Kaplan–Meier survival curves for MEDSS of 3.0 (measured from onset) based on the affected topography at onset.

Kaplan–Meier analysis tested the associations between the progression periods of PwMS, from onset to MEDSS = 3.0 and 6.0, and demographic and clinical factors. The median survival time until MEDSS = 3.0 was 15 years for females and 14 years for males (*p* = 0.39) and 16 and 11 years for patients from urban and rural areas, respectively (*p* = 0.01). We performed Kaplan–Meier analysis to assess survival curves (for MEDSS = 3.0) based on the onset topography. The primary endpoint was the time it took for the patients to reach an EDSS score of 3.0 from the onset of the disease (Table 4). The Log–Rank test (Mantel Cox) indicated a significant difference between the survival curves (*p* < 0.001), suggesting that the type of onset is associated with distinct survival times in PwMS with different disease onset types. Graphical representations are displayed in Figure 2 and Figure 3.

#### 3.2.2. Kaplan–Meier Survival Analysis for MEDSS = 6.0

**Figure 4:**
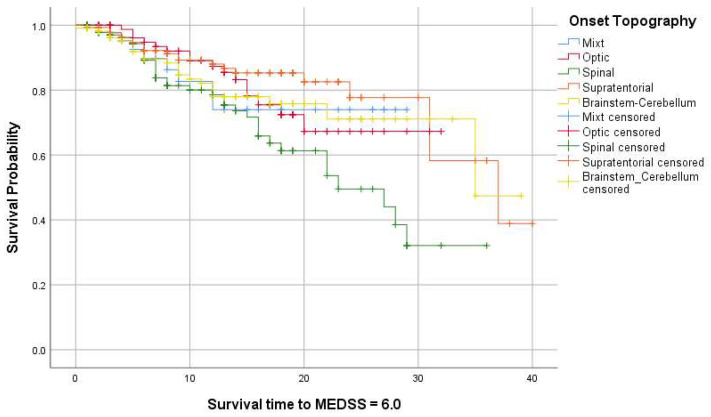
Kaplan–Meier survival curves for MEDSS of 6.0 (measured from onset) based on the onset topography.

The summary of Kaplan–Meier survival analysis for an MEDSS = 6.0 is presented in Table 5. In cases where the survival curves did not reach 50%, the median survival time could not be estimated from the reported data; therefore, the restricted mean and 95% CI were reported for observational purposes. No statistically significant results were reported for gender and environment. Regarding onset topography, the log–rank test (Mantel-Cox) was statistically significant (*p* = 0.20) (Figure 4).

**Figure 5:**
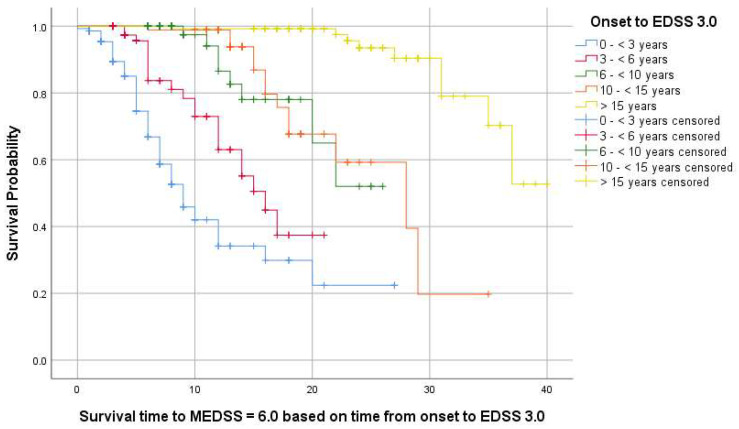
Kaplan–Meier survival curves for MEDSS of 6.0 (measured from onset) based on the time from onset to EDSS of 3.0.

Additionally, we performed Kaplan–Meier analysis to assess survival curves for MEDSS = 6.0, considering the duration from onset to EDSS 3.0. The log–rank test (Mantel–Cox) was statistically significant (*p* < 0.001). In cases where the survival curves did not reach 50%, the median survival time could not be estimated from the reported data; therefore, the restricted mean and 95% CI was reported for observational purposes. Data are reported in Table 6 and Figure 5.

### 3.3. COX Regression Analysis for MEDSS = 3.0 and 6.0

The objective was to assess the association between clinical and demographic factors and the hazard of reaching an EDSS score of 3.0 and 6.0 in PwMS. In the results of our first COX regression analysis examining the hazard of reaching an EDSS score of 3.0 from the onset of MS, for the univariate analysis, gender did not show a significant association with the hazard of reaching an EDSS score of 3.0 (HR = 1.16, 95% CI: [0.86; 1.44], *p* = 0.40). However, the environment had a significant impact, with individuals in rural areas (rural vs. urban) exhibiting a higher hazard (HR = 1.37, 95% CI: [1.05; 1.75], *p* = 0.018). Age at onset demonstrated a strong association with the hazard (HR = 1.05, 95% CI: [1.03;1.06], *p* < 0.001). When considering the onset topography, the Omnibus test showed a statistically significant value (*p* < 0.001). The topography of disease onset revealed significant associations in univariate analysis. Notably, mixt and spinal involvement exhibited increased hazards compared to supratentorial onset (Mixt: HR = 1.82, 95% CI: [1.17; 2.82], *p* = 0.007; Spinal: HR = 1.65, 95% CI: [1.19; 2.29], *p* = 0.003). On the other hand, optic onset did not show a significant association in either analysis. EDSS_0 was a robust predictor of the hazard in both univariate and multivariate analyses (Univariate: HR = 1.73, 95% CI: [1.61; 1.85], *p* < 0.001; Multivariate: HR = 1.71, 95% CI: [1.58; 1.84], *p* < 0.001). R_0 did not demonstrate a significant association with the hazard in either analysis (Table 7).

The second model of COX regression analysis was used to assess the association between clinical and demographic factors in PwMS and the hazard of reaching an EDSS score of 6.0 points. Gender and environment did not reveal any significant associations with the hazard. Age at onset demonstrated a significant association (HR = 1.04, 95% CI: [1.02; 1.06], *p* < 0.001). Onset topography revealed notable associations in univariate analysis, where spinal involvement demonstrated increased hazards compared to supratentorial onset (HR = 2.38, 95% CI: [1.38; 4.10], *p* = 0.002). EDSS_0 was statistically significant for both univariate and multivariate analyses (Univariate: HR = 1.94, 95% CI: [1.73; 2.17], *p* < 0.001; Multivariate: HR = 1.94, 95% CI: [1.72; 2.19], *p* < 0.001). R_0 did not demonstrate a significant association with the hazard in either analysis (Table 8).

## 4. Discussion

The study sample included 523 adults diagnosed with MS treated with DMTs within our clinic. The specific impact of each treatment on disease progression is not within the scope of our study. No significant reduction in relapse rate was noted upon the introduction of immunomodulatory treatment to the entire patient group, but this may be explained by the significant variability within the studied population. This result aligns with the recent findings of Kappos et al., where the authors demonstrated that in RRMS, up to 90% of disease progression occurs independently of relapses [19]. Analyzing relapse rates in patients with stable RR form at the study inclusion, a statistically proven reduction after DMT initiation was observed. In 38% of cases, patients remained relapse-free after treatment initiation. Similar results were reported by Kantor et al. regarding the proportion of relapse-free patients after therapy initiation. The presence of relapses after starting immunomodulatory treatment serves as a negative prognostic clinical biomarker, necessitating a change to a high-efficacy treatment line [38]. A recent study demonstrated that even low-impact relapses in the first two years following diagnosis can nonetheless associate with disease progression, compared to individuals who were relapse-free [39]. 38% of included patients required changing the immunomodulatory treatment line during the reported follow-up (to another first-line) due to clinical and imaging activity (recurrences, new lesions on brain or spinal MRI), progression, adverse reactions, or preference. The study did not account for cases of breakthrough disease when the patients were escaladed to highly active DMTs.

For many individuals with MS who have undergone long-term treatment with conventional, first-line DMTs, the decision to continue the same medication regimen often reflects a personal choice. Despite these DMTs being categorized as first-line platform agents, they effectively control disease activity [40]. Over time, patients developed a sense of stability and routine with their current DMTs. Additionally, the prospect of switching to alternative therapies may evoke concerns about potential risks and side effects, prompting many patients to prioritize the perceived safety and efficacy of the current treatment [41]. The story of first-line therapies in MS is far from over as they continue to demonstrate value and potential in disease management. With their well-established role and efficiency in managing MS symptoms and disease progressions, these agents remain important in the therapeutic arsenal for MS [42,43,44,45].

According to the Atlas of MS, the prevalence of MS in Romania is estimated to be around 26–50 individuals per 100,000 inhabitants [4], while ancestry studies indicate a prevalence of approximately 76 individuals per 100,000 inhabitants [46]. The study population was homogenous, comprising Caucasian individuals from the same geographic area (central region), with similar exposure to conventional risk factors. Genetic factors account for approximately 30% of the risk associated with developing MS, whereas environmental and lifestyle factors play a predominant role in determining MS risk [46]. Co-infection with the Epstein–Barr virus [47], sun exposure [48], and toxic habits such as smoking [49] are regarded as some of the most significant factors contributing to the development of MS.

A predominance of the female gender is noted. Male PwMS were significantly younger (29 years) compared to their female counterparts (31 years), and these differences persist at the initiation of treatment. The results align with data reported in the literature. In a recent epidemiological analysis from 2022, Romero-Pinel et al. reported a mean onset age of 31 years, with no gender-based differences (data obtained from a cohort of 1622 PwMS) [50]. The majority of PwMS presented with supratentorial or spinal topography, followed by infratentorial and visual symptoms. Motor deficits and sensory disturbances are recognized as the primary onset signs of RRMS [51]. Patients with onset through optic neuritis exhibit a lower EDSS score compared to those with spinal symptoms at onset, a clinical fact explained by the impact of altered ambulatory function secondary to spinal involvement [52]. It is acknowledged that onset symptoms involving sensory or visual functions represent a positive prognostic factor regarding disability accumulation [31].

Most of our study’s patients had a considerable disease duration and a treatment duration of over 10 years. In Romania, the first immunomodulatory treatment for MS, specifically subcutaneous administration of interferon beta-1b every two days, was approved in 2000, with the initial administration taking place at Neurology Clinic 1 of the Emergency County Clinical Hospital in Targu Mures. At that time, the rapid initiation of treatment after diagnosis was constrained by prevailing health regulations, which mandated the analysis of patient files and therapy approval based on economic considerations. Since 2014, the administration system for immunomodulatory medications has centralized, providing autonomy to each diagnostic and treatment center, facilitating the early initiation of therapy [53,54]. In over 50% of cases, immunomodulatory treatment was initiated in the same year as the diagnosis, but only in 20% of cases was it initiated in the same year as onset. By comparing the RR to SP population, in our cohort, various time points, including age at onset, DMT initiation, disease duration, onset to treatment, and onset to diagnosis, favor the RRMS individuals. Moreover, the persistence of relapses during DMT administration was higher in RR compared to SP patients.

Kaplan–Meier survival analysis revealed that, within 14 years from onset, half of the monitored PwMS reached an EDSS score of 3.0. The results align with recent data reported in the literature. Scott et al., in a cohort of 184 American treated PwMS followed longitudinally for at least 14 years, demonstrated that within 10.7 years from onset, half of the patients reached an EDSS score of 3.0 [55].

Harari et al., in an epidemiogenetical study that spanned over 18 years and involved a cohort of 2396 Jewish PwMS (Iraqi-born Jewish patients or Israeli-born Jewish patients with both parents born in Iraq vs. those of Northern European origin, who were partially treated with DMTs), concluded that patients of Iraqi origin tended to progress faster to an EDSS of 3.0 compared to those of Northern European origin. This emphasizes ethnic disparities in RRMS progression, although approximately half of the subjects from both cohorts reached an EDSS of at least 3.0—findings and methodology consistent with our study [56]. This variability depends on the heterogeneity of PwMS, accessibility to medical services, as well as genetic, socio-economic, and environmental factors. However, if we compare these results to the natural history of the disease, these patients would present an EDSS of 6.0 within 10–15 years from onset (data from a historical cohort comprising 1099 untreated PwMS) [57,58].

No significant differences were identified regarding the gender of patients in reaching an EDSS score of 3.0. Patients residing in urban areas achieved an EDSS of 3.0 within 16 years from onset, compared to those in rural areas. Poor awareness of onset symptoms, possibly lower levels of education, or delayed access to basic medical services may influence this finding, but further studies are necessary for confirmation. Regarding CNS topography at onset, both spinal and mixed-onset are associated with a rapid accumulation of disability, reaching an EDSS score of 3.0 within 9 years from onset. Spinal onset and polysymptomatic onset are reported as negative prognostic factors in the disease’s progression [59].

Another aspect we investigated in this study was the analysis of the time to reach an EDSS score of 6.0 from onset, specifically within 35 years. There are no significant differences based on gender or place of origin. While natural history studies suggested these values to be between 10–15 years [58], we observe a significant increase with the administration of immunomodulatory treatment, once again emphasizing the importance of the therapeutic window concept [60]. Depending on the stratification from onset to reaching an EDSS of 3.0, a linear increase in the time to conversion to an EDSS of 6.0 is observed. Thus, the longer the duration of conversion to an EDSS of 3.0, the more pronounced the delay in the accumulation of locomotor disability. Patients who took at least 15 years to convert to an EDSS of 3.0 will reach an EDSS of 6.0 in 36 years from onset. These findings account only for patients with an RRMS phenotype at onset and DMT initiation and demonstrate that, despite the persistence of relapses, seeing as how in the studied cohort, the relapse rate was not significantly reduced after DMT initiation, tackling inflammation early in the disease seems to prevent disability accumulation and reduce its impact.

Weinshenker et al., in studies on the natural course of the disease (referring to historical cohorts before the DMT era), found that approximately 50% of PwMS develop a progressive form within 10 years of onset, with the majority reaching an EDSS of 6.0 in the first 15 years of progression [57]. A 2001 study tracking the natural course of 1000 PwMS from the same geographic area calculated average times to irreversible EDSS scores based on the number of relapses and stratified the risk of reaching EDSS of 3.0 and 6.0 accordingly. Thus, two to four relapses will lead to an irreversible EDSS of 3.0 in 8 years and 6.0 in 14–17 years, while five or more relapses will lead to an EDSS of 6.0 in less than 7 years [61]. Tremlett et al. reported the average time to reach an EDSS score of 6 as between 15 and 32 years (data obtained from six patient cohorts, collected from 1972–2004, totaling over 10,000 individuals) and an average time between 15 and 22 years for conversion to a progressive form of the disease (data obtained from three patient cohorts, from 1976–2003, totaling 6565 PwMS) [62].

Following univariate and multivariate COX analyses, the focus was on establishing associations between clinical and demographic parameters and the risk of reaching an EDSS of 3.0 and 6.0. We demonstrated that patients from rural areas have a 37% higher risk compared to urban patients of reaching an EDSS score of 3.0, indicating a long-term need for prioritized efforts in raising awareness about this condition and educating on early symptom recognition to prevent delayed diagnosis. An older age at disease onset is a negative prognostic parameter for irreversible disability scores. An increase of one year at the onset leads to an approximately 5% increase in the risk. This could be partially explained by immunosenescence processes that inevitably lead to a decline in immune response and the endogenous ability to repair damaged myelin, as well as the acceleration of neurogenerative processes that occur physiologically with age [63]. The study participants had an average age of over 31 years at onset, and an age over 30 is considered by many authors as a negative prognostic factor. In two heterogenous cohorts consisting of 855 PwMS (re-evaluated between 2007 and 2010), Tutuncu et al. demonstrated that disease progression acts in an age-dependent manner, where more than half of the included participants transitioned to SPMS by the age of 75 [15]. No data are available regarding the treatment for the two cohorts.

Regarding the topography of CNS involvement, those with infratentorial involvement have a 68% higher risk of reaching an EDSS of 3.0 compared to those with strictly supratentorial involvement. Similarly, those with involvement in multiple CNS areas have an 86% higher risk of reaching an EDSS of 3.0 compared to those with isolated supratentorial onset. The EDSS score recorded at treatment initiation is also a predictive factor for disability accumulation. Multivariate analysis demonstrates that, among the listed parameters, environment, age at onset, and disability level recorded at the initiation of treatment are important predictive factors for reaching an EDSS of 3.0.

Reaching an EDSS score of 6.0 signifies significant locomotor handicap, requiring unilateral assistance for mobility, with important socio-economic repercussions. Multivariate analysis demonstrates that age at disease onset and disability score at treatment initiation are predictive factors for EDSS = 6.0. Onset with symptoms impacting locomotion or function (spinal onset) is associated with a negative prognosis compared to onset through non-locomotor impact symptoms. Although the absolute number of relapses before treatment initiation was not demonstrated as a predictive factor for disability accumulation, they can be indirectly reflected in the EDSS score recorded before treatment initiation. Currently available immunomodulatory therapies, in terms of their mechanism of action, involve inflammation control, and clinically, their effects are reflected in reducing the relapse rate.

However, this is the first broad epidemiological study to assess the progression of PwMS from the same geographical area in our country. Epidemiological studies allow for in-depth analysis of the demographic characteristics of PwMS. As a chronic condition with significant socio-economic impact, epidemiological research can serve as the foundation for developing public health policies to improve the quality of medical services, accessibility, and early identification of these patients. Furthermore, early identification of patients with high progression risk discourages exposure to conventional, first-line DMTs. Machine learning algorithms that could identify signs of progression early based on clinical and paraclinical data (imaging studies, biomarkers of inflammation and neurodegeneration, etc.) emerge as significant tools for modern-day disease management [64,65,66].

Limitations of the presented study include the lack of analysis of the absolute number of relapses in the first 3–5 years from the onset of the disease, and insufficient data regarding relapse severity. This is due to a significant number of patients having a disease duration of over 20 years and historical data not being centralized at that time. Additionally, we did not include the associated comorbidities for the included patients, such as smoking status or alcohol use, nor did we consider participation in rehabilitation programs.

## 5. Conclusions

Our study reinforces the enduring role of conventional first-line DMTs in managing MS progression. Despite a lack of significant reduction in overall relapse rates, stable RRMS patients experienced statistically significant reductions post-DMT initiation. Timely intervention remains crucial in altering the disease trajectory. Despite clinical variability within the studied population, early inflammation control through DMTs may mitigate disability accumulation. Multivariate analysis identified several predictive factors for disability accumulation, including environment, age at onset, and disability level at treatment initiation.

## Figures and Tables

**Table 1:** Clinical and demographic characteristics of the study participants.

Variable	All PwMS (*n* = 523)		Female (*n* = 356)		Male (*n* = 167)			Urban (*n* = 359)		Rural (*n* = 164)		
	Median (IQR)	95% CI	Median (IQR)	95% CI	Median (IQR)	95% CI	*p* #	Median (IQR)	95% CI	Median (IQR)	95% CI	*p* #
Age at study inclusion (years)	46 (37–54)	[44.95; 46.88]	47 (39–55)	[45.59; 47.87]	44 (35–52)	[42.39; 45.97]	0.01 *	47 (39–54)	[45.49; 47.74]	45 (34.75–54.25)	[42.38; 46.23]	0.05
Age at MS onset (years)	31 (25–39)	[31.27; 32.87]	31 (25–40)	[31.60; 33.52]	29 (24.5–36)	[22.59; 32.48]	0.03 *	31 (25–39)	[31; 32.94]	31 (25–39.5)	[30.85; 33.74]	0.67
Age treatment start (years)	35 (28–43)	[35.18; 36.84]	36 (29–43)	[35.54; 37.52]	33 (27–42)	[33.37; 36.44]	0.03 *	35 (29–43)	[35.10; 37.10]	35 (27–43)	[34.31; 37.34]	0.89
Disease duration (years)	13 (7–19)	[13.09; 14.59]	13.5 (7–20)	[13.27; 15.06]	11 (6–19)	[11.77; 14.52]	0.12	14 (7–20)	[13.73; 15.55]	11 (5–17.25)	[10.80; 13.39]	<0.001 *
Treatment duration (years)	9 (4–16)	[9.34; 10.46]	10 (4–16)	[9.52; 10.87]	8 (4–15.5)	[8.29; 10.27]	0.14	10 (4–17)	[9.82; 11.21]	8 (4–13.25)	[7.66; 9.46]	0.003 *
ARR_0	0.08 (0.03–0.22)	[0.18; 0.24]	0.08 (0.31–0.21)	[0.16; 0.23]	0.08 (0.03–0.23)	[0.17; 0.29]	0.65	0.07 (0.03–0.22)	[0.17; 0.26]	0.09 (0.03–0.20)	[0.15; 0.23]	0.30
ARR_1	0.07 (0–0.25)	[0.15; 0.20]	0.08 (0–0.22)	[0.13; 0.21]	0.06 (0–0.31)	[0.14; 0.22]	0.42	0.08 (0–0.25)	[0.15; 0.22]	0.05 (0–0.25)	[0.12; 0.19]	0.38
ARR_T	0.28 (0.16–0.50)	[0.36; 0.42]	0.26 (0.15–0.5)	[0.33; 0.40]	0.33 (0.16–0.5)	[0.31; 0.49]	0.06	0.26 (0.15–0.50)	[0.34; 0.41]	0.33 (0.17–0.56)	[0.36; 0.48]	0.08
EDSS_0	2 (1–3)	[2.14; 2.39]	2 (1–3.12)	[2,15; 2.46]	2 (1–3)	[1.95; 2.39]	0.32	2 (1.5–3.0)	[2.08; 2.38]	2 (1–3.5)	[2.11; 2.57]	0.42
EDSS_1	3 (1.5–4.5)	[2.99; 3.34]	2.75 (1.5–4.5)	[2.96; 3.39]	3 (1–4.75)	[2.84; 3.45]	0.86	2.5 (1.5–4.5)	[2.91; 3.34]	3.0 (1.5–4.5)	[2.94; 3.55]	0.41

# Non-parametric Mann–Whitney *U* test; ARR: annual relapse rate (ARR_0: relapse rate before treatment, ARR_1: relapse rate upon treatment, ARR_T: total relapse rate); EDSS: expanded disability status scale (EDSS_0: at the patient’s first visit, EDSS_1: upon inclusion in the study). Variable values are reported as median and IQR, 95% CI; * statistically significant.

**Table 2:** Clinical and demographical characteristics of RRMS and SPMS patients.

Variable	RRMS (*n* = 436)		SPMS (*n* = 87)		*p* *
Environment (Urban:Rural)	293:143		66:21		0.07
Gender (F:M)	297:139		59:28		0.52
	Median (IQR)	95% CI	Median (IQR)	95% CI	*p* #
Age at study inclusion (years)	44.50 (35.75–52)	[43.13; 45.17]	54 (48.50–62)	[52.90; 56.61]	<0.001 *
Age at MS onset (years)	30 (24–39)	[30.64; 32.37]	33 (28–40)	[32.87; 36.97]	0.001 *
Age treatment start (years)	34 (27–42)	[34.12; 35.89]	40 (33–48.5)	[39.03; 43.07]	<0.001 *
Disease duration (years)	12 (6–18)	[11.88; 13.41]	19 (14–24)	[17.92; 21.76]	<0.001 *
Onset to treatment (years)	1.5 (1–5)	[3.04; 3.97]	4 (1–8)	[4.58; 7.67]	<0.001 *
Onset to diagnosis (years)	1 (0–3)	[2.14; 2.99]	1 (0–5)	[2.65; 5.21]	0.03 *
Treatment duration (years)	8 (4–15)	[8.55; 9.74]	15 (8–19)	[12.43; 14.99]	<0.001 *
ARR_0	0.09 (0.03–0.25)	[0.18; 0.25]	0.04 (0.02–0.14)	[0.10; 0.25]	<0.001 *
ARR_1	0.04 (0–0.23)	[0.13; 0.18]	0.17 (0.08–0.30)	[0.18; 0.31]	<0.001 *
ARR_T	0.3 (0.04–0.30)	[0.37; 0.43]	0.23 (0.04–0.23)	[0.26; 0.38]	0.06
EDSS_0	1.5 (1–2.65)	[1.82; 2.05]	4 (3–5)	[ 3.60; 4.24]	<0.001 *
EDSS_1	2 (1–3.5)	[2.41; 2.72]	6 (6–6.5)	[6.00; 6.37]	<0.001 *

* Chi-Square, statistically significant result (*p* < 0.05); # non-parametric Mann–Whitney *U* test; ARR: annual relapse rate (ARR_0: relapse rate before treatment, ARR_1: relapse rate upon treatment, ARR_T: total relapse rate); EDSS: expanded disability status scale (EDSS_0: at the patient’s first visit, EDSS_1: upon inclusion in the study). Variable values are reported as median and IQR, 95% CI.

**Table 3:** Characteristics of the onset topography according to clinical parameters.

	Supratentorial (*n* = 142)	Spinal (*n* = 139)	Infratentorial (*n* = 109)	Optic (*n* = 88)	Mixt (*n* = 45)	*p* ¥	_adjusted_ *p*
ARR_0	0.06 (0.03–0.2)	0.1 (0.04–0.25)	0.10 (0.03–0.28)	0.05 (0.02–0.16)	0.07 (0.28–0.15)	0.01 *	>0.05
ARR_1	0.06 (0–0.24)	0.25 (0–0.25)	0.06 (0–0.25)	0.09 (0–0.25)	0 (0–0.33)	0.94	
ARR_T	0.26 (0.16–0.5)	0.34 (0.18–0.56)	0.26 (0.13–0.5)	0.22 (0.14–0.45)	0.30 (0.25–0.50)	0.04 *	>0.05
EDSS_0	1.5 (1.0–3)	2.5 (1–3.5)	2 (1–3)	1.5 (1–2.5)	3 (1.5–3.5)	<0.001 *	<0.05
EDSS_1	2 (1–4)	3.5 (2–6)	2.5 (1.5–4.0)	2.0 (1.37–3.5)	3.5 (2.5–5)	0.002 *	<0.05

¥ Non-parametric Kruskal–Wallis Test, **_adjusted_***p* Dunn–Bonferroni correction; ARR: annual relapse rate (ARR_0: relapse rate before treatment, ARR_1: relapse rate upon treatment, ARR_T: total relapse rate); EDSS_0: EDSS score at the patient’s first visit; EDSS_1: EDSS score at study inclusion; * statistically significant result (*p* < 0.05).

**Table 4:** Survival time until MEDSS = 3.0 based on clinical and demographic factors.

MEDSS = 3.0		Median Survival Time (Years), 95% CI	pLog-Rank
Patient lot		14 [11.34; 16.66]	
Gender	Female	15 [10.85; 19.15]	0.39
	Male	14 [10.23; 17.76]	
Environment	Urban	16 [12.42; 19.57]	0.015 *
	Rural	11 [7.73; 14.26]	
Onset topography	Supratentorial	19 [13.72; 24.27]	<0.001 *
	Optic	20 [17.08; 23.17]	
	Spinal	9 [1.13; 6.78]	
	Infratentorial	18 [12.86; 23.13]	
	Mixt	9 [7.95; 17.85]	

CI: 95% Confidence interval; * statistically significant result (*p* < 0.05).

**Table 5:** Survival time until MEDSS = 6.0 based on clinical and demographic factors.

MEDSS = 6.0		Median Survival Time (Years), 95% CI	Mean Survival Time (Years), 95% CI	pLog-Rank
Patient lot		35 [30.11; 39.88]		
Gender	Female	35 [29.20; 40.78]		0.59
	Male	-	27.30 [24.6; 29.94]	
Environment	Urban	-	27.66 [27.03; 30.83]	0.41
	Rural	35 [21.34; 48.65]		
Onset topography	Supratentorial	37 [26.1; 47.9]		0.20 *
	Optic	-	25.82 [23.18; 28.46]	
	Spinal	23 [14.95; 31.04]		
	Infratentorial	35 [26.41; 33.17]		
	Mixt	-	23.56 [20.25; 26.83]	

95% CI: 95% Confidence interval; * statistically significant result (*p* < 0.05); mean survival time (years) with 95% CI was reported when median survival time could not be estimated from the reported data.

**Table 6:** Kaplan–Meier analysis for MEDSS = 6.0 based on the duration from onset to EDSS = 3.0.

MEDSS = 6.0	Median Survival Time (Years), 95% CI	Mean Survival Time (Years), 95% CI	pLog-Rank
<3 years	9 [6.93; 11.06]		<0.001 *
3 – <6 years	16 [12.27; 19.72]		
6 – <10 years	-	21.64 [19.19; 24.09]	
10 – <15 years	28 [17.45; 38.54]		
≥15 years	-	36.39 [34.48; 38.39]	

95% CI: 95% Confidence interval; * statistically significant result (*p* < 0.05).

**Table 7:** COX regression analysis for the hazard of reaching an EDSS of 3.0.

Variable	Univariate Analysis		Multivariate Analysis	
	HR. (95% CI)	*p*	HR. (95% CI)	*p*
Gender (M vs. F)	1.16 [0.86; 1.44]	0.40	-	-
Environment (Rural vs. Urban)	1.37 [1.05; 1.75]	0.018 *	1.30 [1.00; 1.68]	0.04 *
Age at onset (years)	1.05 [1.03; 1.06]	<0.001 *	1.04 [1.02; 1.05]	<0.001 *
Onset Topography		<0.001 *		
Infratentorial vs. Supratentorial	1.00 [0.69; 1.45]	0.98	0.86 [0.36; 2.01]	0.73
Mixt vs. Supratentorial	1.82 [1.17; 2.82]	0.007 *	1.23 [0.77; 1.94]	0.37
Optic vs. Supratentorial	0.76 [0.50; 1.61]	0.20	−	−
Spinal vs. Supratentorial	1.65 [1.19; 2.29]	0.003 *	1.12 [0.87; 1.71]	0.24
EDSS_0	1.73 [1.61; 1.85]	<0.001 *	1.71 [1.58; 1.84]	<0.001 *
R_0	1.06 [0.99; 1.36]	0.09	−	−

M: male; F: female; EDSS: expanded disability status scale (EDSS_0: at the patient’s first visit; R_0: relapses before treatment; HR = relative hazard; 95% CI: 95% confidence interval, * statistically significant result (*p* < 0.05).

**Table 8:** COX regression analysis for the hazard of reaching an EDSS of 6.0.

Variable	Univariate Analysis		Multivariate Analysis	
	HR. (95% IC)	*p*	HR. (95% IC)	*p*
Gender (M vs. F)	0.98 [0.64; 1.50]	0.96	-	-
Environment (Rural vs. Urban)	1.18 [0.78; 1.80]	0.42	-	-
Age at onset (years)	1.04 [1.02; 1.06]	<0.001 *	1.04 [1.02; 1.06]	<0.001
Onset Topography		0.03 *		
Infratentorial vs. Supratentorial	1.42 [0.78; 2.59]	0.24	1.24 [0.67; 2.27]	0.48
Mixt vs. Supratentorial	1.47 [0.64; 3.34]	0.35	0.93 [0.49; 2.15]	0.87
Optic vs. Supratentorial	1.33 [0.69; 2.56]	0.39	1.35 [0.69; 2.65]	0.37
Spinal vs. Supratentorial	2.38 [1.38; 4.10]	0.002	1.67 [0.95; 2.93]	0.73
EDSS_0	1.94 [1.73; 2.17]	<0.001 *	1.94 [1.72; 2.19]	<0.001
R_0	1.01 [0.91; 1.13]	0.75	-	-

M: male; F: female; EDSS: expanded disability status scale (EDSS_0: at the patient’s first visit; R_0: relapses before treatment; HR = relative hazard; 95% CI: 95% confidence interval, * statistically significant result (*p* < 0.05).

## Data Availability

Data are contained within the article.
